# Identification of a New Stromal Cell Type Involved in the Regulation of Inflamed B Cell Follicles

**DOI:** 10.1371/journal.pbio.1001672

**Published:** 2013-10-01

**Authors:** Cyrille Mionnet, Isabelle Mondor, Audrey Jorquera, Marie Loosveld, Julien Maurizio, Marie-Laure Arcangeli, Nancy H. Ruddle, Jonathan Nowak, Michel Aurrand-Lions, Hervé Luche, Marc Bajénoff

**Affiliations:** 1 Centre d'Immunologie de Marseille-Luminy (CIML), Aix-Marseille University, UM2, Marseille, France; 2 Institut National de la Santé et de la Recherche Médicale (INSERM), U1104, Marseille, France; 3 Centre National de la Recherche Scientifique (CNRS), UMR7280, Marseille, France; 4 INSERM, U1068, CRCM, Marseille, France; 5 CNRS, UMR7258, CRCM, Marseille, France; 6 Aix-Marseille Univ, F-13284, Marseille, France; 7 Institut Paoli-Calmettes, Marseille, France; 8 Yale University School of Medicine, New Haven, Connecticut, United States of America; University of Pennsylvania, United States of America

## Abstract

Lymph node (LN) stromal cells provide survival signals and adhesive substrata to lymphocytes. During an immune response, B cell follicles enlarge, questioning how LN stromal cells manage these cellular demands. Herein, we used a murine fate mapping system to describe a new stromal cell type that resides in the T cell zone of resting LNs. We demonstrated that upon inflammation, B cell follicles progressively trespassed into the adjacent T cell zone and surrounded and converted these stromal cells into CXCL13 secreting cells that in return delineated the new boundaries of the growing follicle. Acute B cell ablation in inflamed LNs abolished CXCL13 secretion in these cells, while LT-β deficiency in B cells drastically affected this conversion. Altogether, we reveal the existence of a dormant stromal cell subset that can be functionally awakened by B cells to delineate the transient boundaries of their expanding territories upon inflammation.

## Abstract 2

Identification of a new stromal cell type in mouse lymph nodes that can be activated by B cells to delineate the transient boundaries of B cell zones during inflammation

## Author Summary

Immune responses develop in lymphoid organs such as the tonsils and lymph nodes (LNs), which are composed of leukocytes (95%) and architectural stromal cells (5%). LNs involved in mounting an immune response recruit large numbers of lymphocytes and support the division of those that recognise the foreign antigen, raising the question of how LN stromal cells manage this tremendous remodeling. In this study, we focused on specific zones within the lymph node called germinal centres that comprise dense aggregates or follicles of B lymphocytes, and investigated how lymphoid stromal cells contribute to the reorganization of primary B cell follicles into large reactive secondary follicles. Using a fate mapping system in mice, we identified a new stromal cell type that resides in the T cell zone of noninflamed resting LNs. We demonstrate that upon inflammation, B cells usually contained within B cell follicles progressively trespass into the adjacent T cell zone and surround and convert resident stromal cells into cells that can secrete CXCL13, a B cell chemokine. These CXCL13-secreting cells in turn act to delineate the new transient boundaries of the growing follicle. Identification of this distinct versatile stromal cell type adds to our understanding of mechanisms underlying compartmentalization of lymphoid organs into their functional zones.

## Introduction

In a LN, fibroblastic reticular cells (FRCs) reside in the T cell zone, while follicular dendritic cells (FDCs) populate B cell follicles [1]–[3]. Both stromal cell populations form dense, intermingled three-dimensional networks that provide survival signals to lymphocytes and create adhesive substrata on which these cells actively migrate [1],[4]–[7]. FRCs and FDCs, respectively, secrete the homeostatic chemokines CCL19/CCL21 and CXCL13 [7],[8]. Because the secondary lymphoid organs (SLOs) of CCL19/CCL21- and CXCL13-deficient animals display profoundly altered T and B cell areas, this segregated production of chemokines is thought to control the localization and size of T and B cell zones in resting SLOs [8],[9]. To date, these two stromal cell types are the only ones that have been reported to regulate T and B cells territoriality and migration within SLOs [3],[4],[10].

While resting LNs continuously host and nourish a fixed number of lymphocytes, inflamed LNs massively recruit naive lymphocytes and support the division of the antigen-specific ones [11]–[13]. These combined events induce a drastic enlargement of inflamed LNs, raising the question of how the stromal cells present in such LNs manage to host, nourish, and guide this increased amount of cells.

Herein, we have focused our attention on B cell follicles, seeking to understand how stromal cells accommodate the progressive growth of these structures in inflamed LNs. To this aim, we tracked the fate of murine complement receptor 2 (CD21)–expressing stromal cells and discovered a new stromal cell type involved in the regulation of B cell follicle growth.

## Results

### Identification of a New Lymphoid Stromal Cell Type

**Figure 1:**
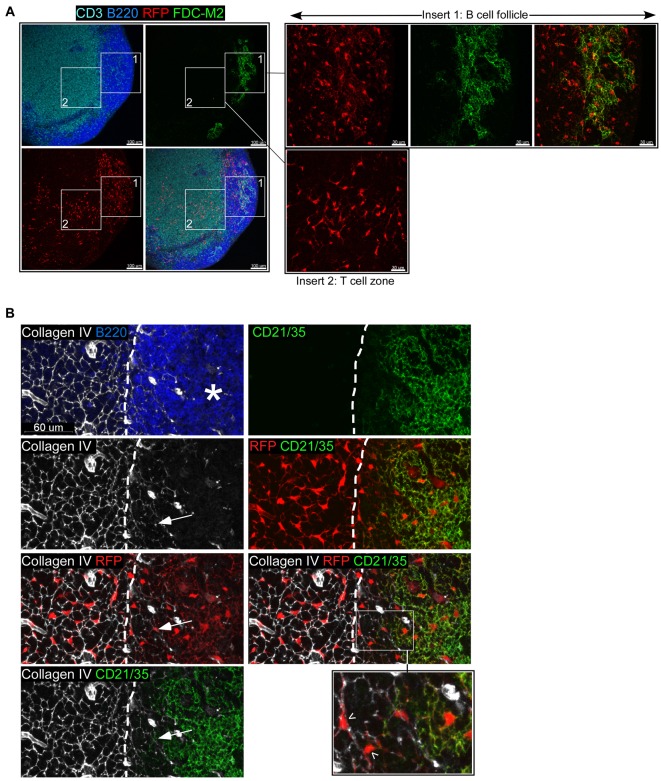
Visualization of a new subset of LN stromal cells. Confocal images of LN sections from a CD21cre-RFP chimera stained for (A) FDC-M2 (green), CD3 (light blue), and B220 (dark blue) and (B) CD21/35 (green), Collagen IV (white), and B220 (blue). RFP^+^ cells appear in red. In (B), the star (*) signals the position of the collagen-poor central region of the follicle populated by the CD21^+^ RFP^+^ FDC network; arrows indicate the extensions of the conduit network within the follicle and arrowheads point to CD21^−^ RFP^+^ cells attached to these conduits. The dashed line represents the delineation of the B220 staining. No RFP signal was detected in CD21cre^−^RFP^+^ chimeric mice, ruling out a leaky expression of the reporter. Data are representative of three different experiments (two mice per experiment).

FDCs regulate the survival, territory, and migration of naive B cells in noninflamed SLOs [1],[4],[6],[10]. Upon an immune response, these stromal cells also support the proliferation and selection of activated B cells in germinal centers (GCs). We thus assumed that FDCs could regulate the growth of B cell follicles in inflamed LNs. In order to track these stromal cells during an immune response, we generated a mouse in which FDCs would be fluorescently and permanently labeled. To this aim, we crossed CD21-cre mice to the inducible Rosa-tdRFP mice (tandem dimer red fluorescent protein). In the CD21-cre mouse, the cre recombinase is placed under the control of the mouse complement receptor 2 (CD21) promoter, while in the Rosa-tdRFP mouse, the expression of the tdRFP is blocked by a *loxP*-flanked STOP fragment placed between the *Gt(ROSA)26Sor* promoter and the tdRFP sequence [14],[15]. In this double transgenic (Tg) mouse, any cell that expresses (or has expressed) CD21 permanently produces RFP. In mice, CD21 is primarily expressed on mature B lymphocytes and FDCs [16],[17]. In order to generate a mouse in which radiation-resistant FDCs, but not radiation-sensitive B cells, would express RFP, we irradiated these mice and reconstituted them with Wt bone marrow cells [10]. Eight weeks after bone marrow transfer, the LNs of these CD21cre-RFP chimeras were sectioned; stained with fluorescent anti-FDC, –T cell, and –B cell Abs; and analyzed by confocal microscopy. As expected, in the LNs of these chimeric mice, FDC-M2^+^ (complement C4) FDCs expressed RFP in B cell follicles (Figure 1A, insert 1). Surprisingly, a second population of RFP^+^ cells was consistently present in the T cell zone of these LNs (Figure 1A, insert 2). Unlike FDCs, these stromal cells were negative for CD21 expression despite their expression of RFP and attached to the dense collagenous conduit network that defines the territory of the T cell zone and its extensions within the inner border of B cell follicles (Figure 1B and [4],[18]). Interestingly, these cells were still present in the LNs of nonirradiated CD21cre-RFP mice crossed to B cell–deficient mice (μMT, Figure S1A), and RAG-2^−/−^ mice (Figure S1B), indicating that unlike FDCs, these cells did not require B cells to develop and that their presence did not result from the irradiation protocol used to generate bone marrow chimeras [19],[20].

### CD21^−^ RFP^+^ Are Not Precursors of FDCs, MRCs, or Conventional FRCs

As CD21^−^ RFP^+^ cells and FDCs were the only cell types expressing RFP in the LNs of CD21cre-RFP chimeras, we reasoned that they could be somehow related. B cell–deficient mice lack FDC networks that are fully reconstituted upon B cell adoptive transfer via the differentiation of FDC precursors [21]. Therefore, the “new” subset of RFP^+^ stromal cells described above may equal those FDC-precursors. In order to test this hypothesis, we first adoptively transferred Wt B cells into nonirradiated CD21cre-RFP mice crossed to μMT B cell–deficient mice. One day later, the location of the transferred B cells was assessed on LN tissue sections. Confocal microscopy indicated that naive B cells did not spread in the whole LN but aggregated in clusters at the periphery of the LNs, precisely where primary follicles developed a few days later [22] (Figure S1C). Therefore, we concluded that the RFP^+^ stromal cells located in the T cell zone were unlikely to be the conventional FDC precursors responsible for the development of B cell follicles.

**Figure 2:**
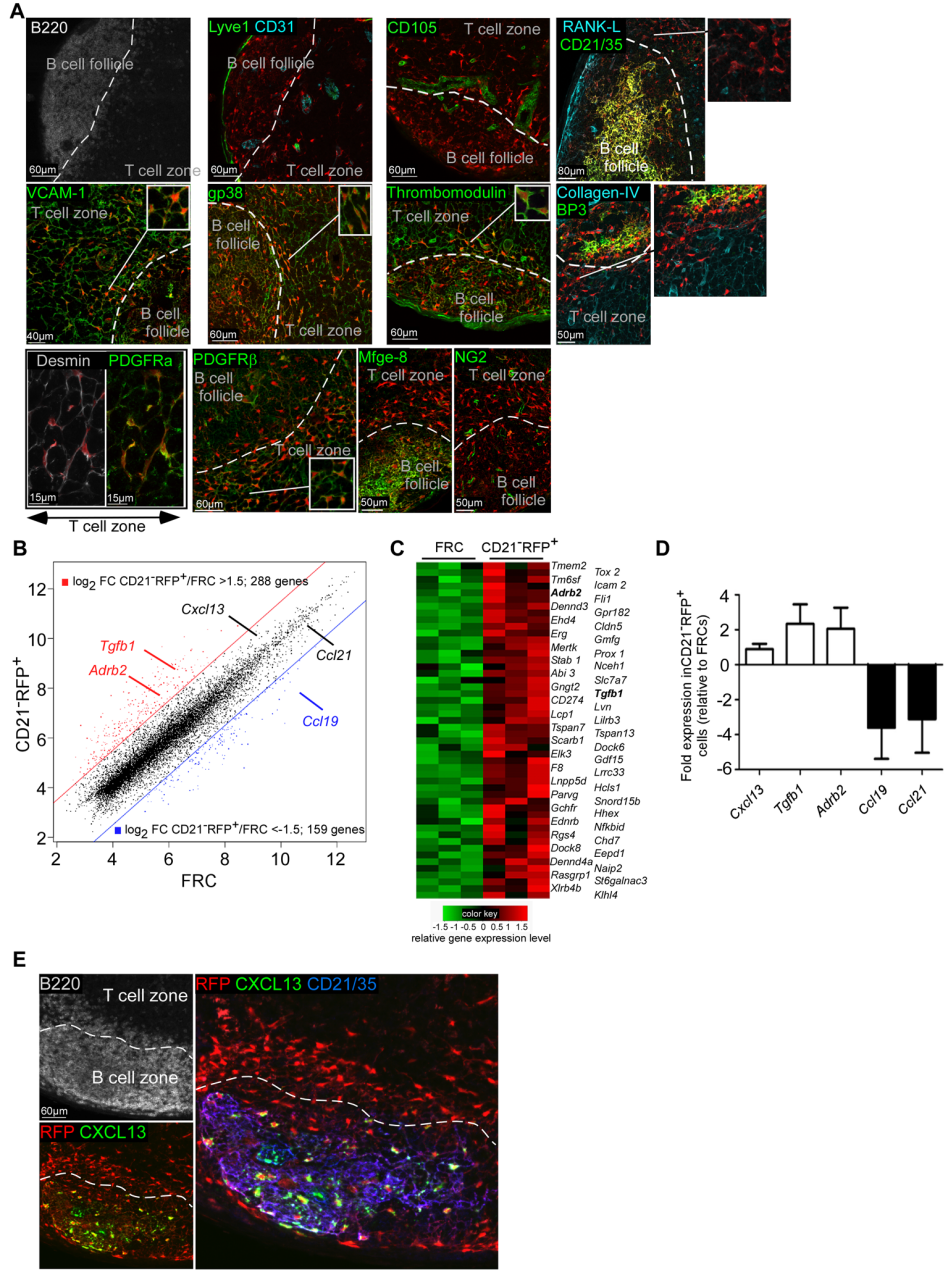
Phenotype of CD21^−^ RFP^+^ stromal cells. (A and E) Confocal images of a LN section from a CD21cre-RFP chimera stained for the indicated markers. RFP^+^ cells appear in red. The dashed line delineates B cell follicle boundary. (B, C, D) CD21^−^ RFP^−^ gp38^+^ CD31^−^ CD45^−^ FRCs and CD21^−^ RFP^+^ gp38^+^ CD31^−^ CD45^−^ cells were sorted by flow cytometry and their transcriptomic profiles were analyzed by microarrays (B and C) or RT-PCR (D). (B) Scatter plot of global comparison of gene expression between CD21^−^ RFP^+^ cells and FRCs. Each gene in the microarray is represented by a dot with coordinates consisting of average gene expression computed from three independent CD21^−^ RFP^+^ samples (*y*-axis) and from the three matched FRC samples (*x*-axis). Genes with Log_2_ average expression level at least 1.5-fold higher in CD21^−^ RFP^+^ cells are shown in red, while genes with Log_2_ average expression level at least 1.5-fold higher in FRCs are shown in blue. These genes are separated from the other genes by colored lines representing these fold change cutoffs. (C) Heat map for the 50 genes with the most significantly higher expression in CD21^−^ RFP^+^ cells. A subset of the genes shown in red in panel B was further selected based on a *p* value<0.05 for differential expression between CD21^−^ RFP^+^ cells and FRCs, as computed by Limma. Genes (rows) and samples (columns) were clustered by complete linkage hierarchical clustering, using Euclidean distance measure. For each gene, expression levels close to the mean value across all six samples are shown in black, high expression levels in red, and low expression levels in green. (D) Relative expression of *Cxcl13*, *Ccl21*, *Ccl19*, *Tgfb1*, and *Adrb2* mRNA levels quantified by RT-PCR. Data are representative of three different experiments (4–5 mice per sample). (E) Confocal images of a LN B cell follicle and its adjacent T cell area from a CD21cre-RFP chimera stained for CXCL13 (green), CD21/35 (blue), and B220 (white). RFP^+^ cells appear in red. The dashed line represents the delineation of the B220 staining. Data are representative of two different experiments (two mice per experiment).

Krautler et al. recently showed that FDCs arise from a subpopulation of stromal cells called preFDC that (i) can be found in the T cell zone of lymphoid organs, (ii) lack markers of mature FDCs such as CD21 and FDC-M2, and (iii) express NG2, milk-fat globule epidermal growth factor 8 (Mfge8), and platelet-derived growth factor receptor β (PDGFRβ) [23]. We then performed additional immunostainings to determine if CD21^−^ RFP^+^ cells were preFDCs. Confocal microscopy revealed that RFP^+^ stromal cells located in the T cell zone of CD21cre-RFP LNs as well as in the inner B cell follicle border were CD21^−^ FDC-M2^−^ CD16/32^−^ CD31^−^ Aire^−^ CD105^−^ Lyve-1^−^ NG2^−^ BP3^−^ Mfge8^−^ PDGFRβ^+^ PDGFRα^+^ gp38^+^ thrombomodulin^+^ desmin^+^ Vcam-1^+^ and co-existed with gp38^+^ thrombomodulin^+^ PDGFRβ^+^ PDGFRα^+^ desmin^+^ FRCs (Figure 2A, Figure S2, and unpublished data). These markers indicated that CD21^−^RFP^+^ cells were not preFDCs, follicular stromal cells, or blood or lymphatic endothelial cells.

Marginal Reticular Cells (MRCs) are poorly defined stromal cells thought to descend from organizer stromal cells in the anlagen [24]. MRCs are located immediately below the subcapsular sinus and express Receptor Activator of Nuclear factor Kappa-β ligand (RANK-L) [24]. Immunostaining of CD21cre-RFP chimeric LNs for RANK-L expression showed that CD21^−^ RFP^+^ stromal were RANK-L negative and were thus not MRCs (Figure 2A).

Anatomical and phenotypic analysis indicated that FRCs and CD21^−^ RFP^+^ stromal cells only differed by their ontogenic expression of *CD21*. Therefore, one could wonder whether CD21^−^ RFP^+^ stromal cells represented a new stromal cell type or an unconventional subset of FRCs. In order to address this issue, we performed a genome-wide transcriptional profiling of purified FRCs and CD21^−^ RFP^+^ stromal cells. Microarray analysis indicated that FRCs and CD21^−^ RFP^+^ stromal cells differed by the expression of a total of 447 genes based on a log_2_ fold change (FC) cutoff set at 1.5 (Figure 2B). This suggested that CD21^−^ RFP^+^ cells broadly differed from conventional FRCs and represented a distinct type of stromal cells. To more precisely define the specific transcriptomic signature of CD21^−^ RFP^+^ stromal cells as compared to FRCs, fold change patterns of most highly differentially expressed genes in FRCs and CD21^−^ RFP^+^ stromal cells were selected, analyzed, and displayed in a heat map (Figure 2C). The expression of some differentially expressed genes including pivotal soluble factors implicated in various stromal cell–mediated immunological functions was then examined by real-time polymerase chain (RT-PCR) (Figure 2D). CD21^−^ RFP^+^ stromal cells were confirmed to express more *Tgfb1* and beta-2 adrenergic receptor (*Adbr2*) transcripts than FRCs. Conversely, FRCs expressed more *Ccl21* and *Ccl19* transcripts. Both cell types were confirmed to express *Cxcl13* transcripts in comparable amounts.

As FDCs also express CXCL13, we analyzed the ability of CD21^−^ RFP^+^ cells to produce CXCL13 by immunostaining. This approach indicated that only 0.7% (22 out of 3,131) of the CD21^−^ RFP^+^ cells present in the LN T cell zone and the T/B border were positive for CXCL13, while CD21^+^ FDCs displayed strong CXCL13 signals (Figure 2E).

Altogether, these data suggested that CD21^−^ RFP^+^ stromal cells were neither preFDCs, FDCs, follicular stromal cells, MRCs, nor a nonconventional subset of FRCs.

### Inflamed B Cell Follicles Trespass in the T Cell Zone

During an immune response, growing GCs expand in the center of primary follicles, repelling to their borders the numerous naive B cells that the draining LNs continue to recruit [25],[26]. Such a mechanism creates the so-called follicular mantle composed of naive B cells. As the collagenous capsule of the LN is rigid, we reasoned that B cell follicles might grow out of their own zone and trespass in the adjacent T cell zone covered by the conduit system. In this situation, the CD21^−^ RFP^+^ stromal cells located in this area would become part of the follicular mantle and perhaps instructed to display new functionalities. In order to test this hypothesis, we first sought to determine if inflamed B cell follicles invaded the adjacent T cell zone. We injected complete freund adjuvant (CFA) in the ears of Wt mice and analyzed the kinetics of B cell recruitment in their ear draining LNs (dLNs) (Figure S3). Based on these results, we injected mice with CFA and stained their dLNs for CD3, B220, and collagen-IV (or ERTR-7) expression 3 wk later, at the peak of B cell recruitment/proliferation (Figure S4 and unpublished data). As previously described, primary B cell follicles of control LNs were confined to regions containing sparse conduits and blood capillaries (Figure S4, left panel) [18],[22],[27]. On the contrary, we determined that inflamed B cell follicle borders were enriched in conduits, suggesting that inflamed B cell follicles trespassed in the adjacent T cell zone during their growth (Figure S4, right panel).

**Figure 3:**
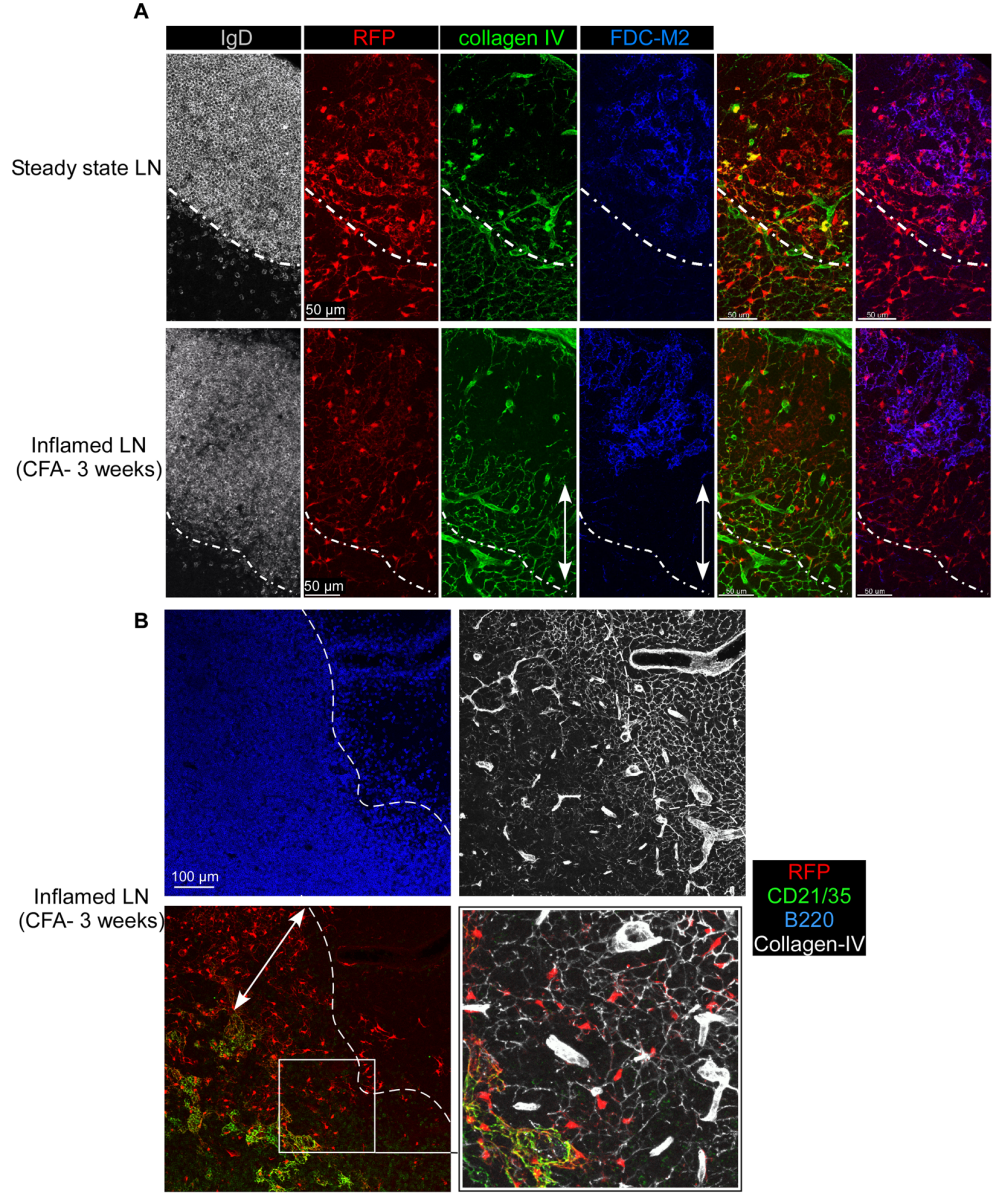
CD21^−^ RFP^+^ stromal cells are surrounded by inflamed B cell follicles. CD21cre-RFP chimeras were untreated (A, upper panel) or injected (A, lower panel and B) with an emulsion of CFA/PBS in the ears. (A) Three weeks later, ear draining LNs were sectioned, stained for collagen IV (green), IgD (white), and FDC-M2 expression (blue) and imaged by confocal microscopy. RFP^+^ cells appear in red. (B) Confocal pictures of an inflamed B cell follicle stained for collagen IV (white), B220 (blue), and CD21/35 (green) expression. Arrows highlight the region of the inflamed B cell follicle enriched in collagen IV. Insert displays high magnification of the boundary between T and B cell areas (dashed line). Data are representative of three different experiments (two mice per experiment).

We performed the same experimental protocol in CD21-cre RFP chimeras and analyzed if the borders of their enlarged follicles contained RFP^+^ stromal cells attached to the conduit system. LN sections from CD21-RFP chimeric mice injected with CFA 3 wk previously were stained with anti-T, -B, and -FDC Abs and analyzed by confocal microscopy (Figure 3 and Figure S5). Using such strategy, we determined that FDC-M2^+^ CD21^+^ PDGFRβ^lo^ RFP^+^ FDCs were still confined to the collagen-poor regions of the enlarged B cell follicles, while the collagen-enriched borders of these follicles contained numerous FDC-M2^−^ CD21^−^ PDGFRβ^hi^ RFP^+^ stromal cells attached to the conduit system (Figure 3 and Figure S5). Altogether, these results strongly suggested that during their enlargement, B cell follicles surrounded the CD21^−^ RFP^+^ stromal cells initially located in the adjacent T cell zone of noninflamed LNs but did not convert them into conventional FDCs.

### B Cells Instruct Surrounded CD21^−^ RFP^+^ Stromal Cells to Secrete CXCL13

**Figure 4:**
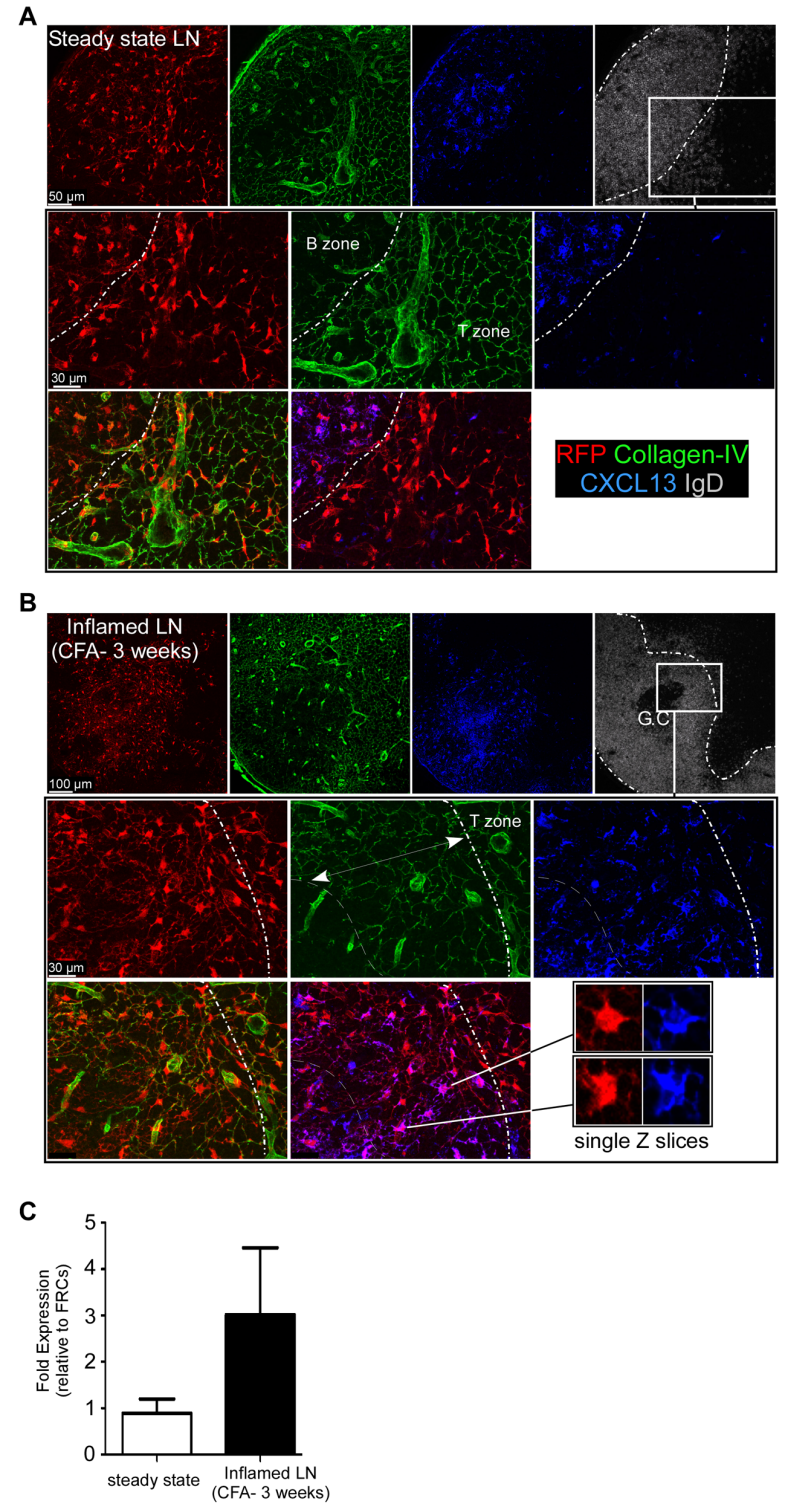
Induction of CXCL13 secretion in CD21^−^ RFP^+^ stromal cells upon B cell follicle enlargement. CD21cre-RFP chimeras were untreated (A) or injected (B) with an emulsion of CFA/PBS in the ears. Three weeks later, ear draining LNs were sectioned, stained for collagen IV (green), IgD (white), and CXCL13 expression (blue), and imaged by confocal microscopy. RFP^+^ cells appear in red. Inserts display high magnifications of the boundaries between T and B cell areas (dashed line). In (B), the arrow indicates the area of the enlarged B cell follicle covered by the collagen IV network (conduit system). Single *z* slices show intracellular staining of CXCL13. (C) CXCL13 mRNA levels in CD21^−^ CD45^−^ RFP^+^ cells purified from resting and CFA-inflamed LNs relative to FRCs. Data are representative of three different experiments (two mice (A and B) and five mice (C) per experiment).

The formation and maintenance of FDC networks as well as their secretion of CXCL13 require their continuous interaction with B cells [1],[8],[20],[28]. We hypothesized that the massive recruitment of B cells at the borders of enlarged follicles combined with the withdrawal of the T cell area may induce the secretion of CXCL13 in the surrounded CD21^−^ RFP^+^ stromal cells. In order to test this hypothesis, we injected CFA in the ears of CD21cre-RFP chimeras, and 3 wk later, their dLNs were sectioned; stained for IgD, collagen-IV, and CXCL13 expression; and analyzed by confocal microscopy (Figure 4). The area of inflamed LNs covered by the conduit system was then divided in two regions according to the presence of T cells or B cells. In the region populated by T cells (true T cell zone), we determined that 4.9% (21 out of 428) of the RFP^+^ stromal cells were positive for CXCL13 staining. On the other hand, in the borders of enlarged B cell follicles densely populated by the conduit system (and thus devoid of FDC-M2^+^ FDCs), we determined that 85.7% (365 out of 426) of RFP^+^ stromal cells displayed an intracellular CXCL13 staining (Figure 4A and B). These results were also confirmed at the mRNA level using RT-PCR on CD21^−^ RFP^+^ cells isolated from resting and inflamed LNs (Figure 4C). Importantly, 97% of CXCL13^+^ cells embedded in the borders of enlarged B cell follicles were CD21^−^ RFP^+^, indicating that conventional CD21^−^ RFP^−^ FRCs did not significantly contribute to CXCL13 secretion in this region (unpublished data).

Based on these evidences, CXCL13^+^ CD21^−^ RFP^+^ stromal cells surrounded by inflamed B cell follicles will hereafter be qualified as “converted.”

**Figure 5:**
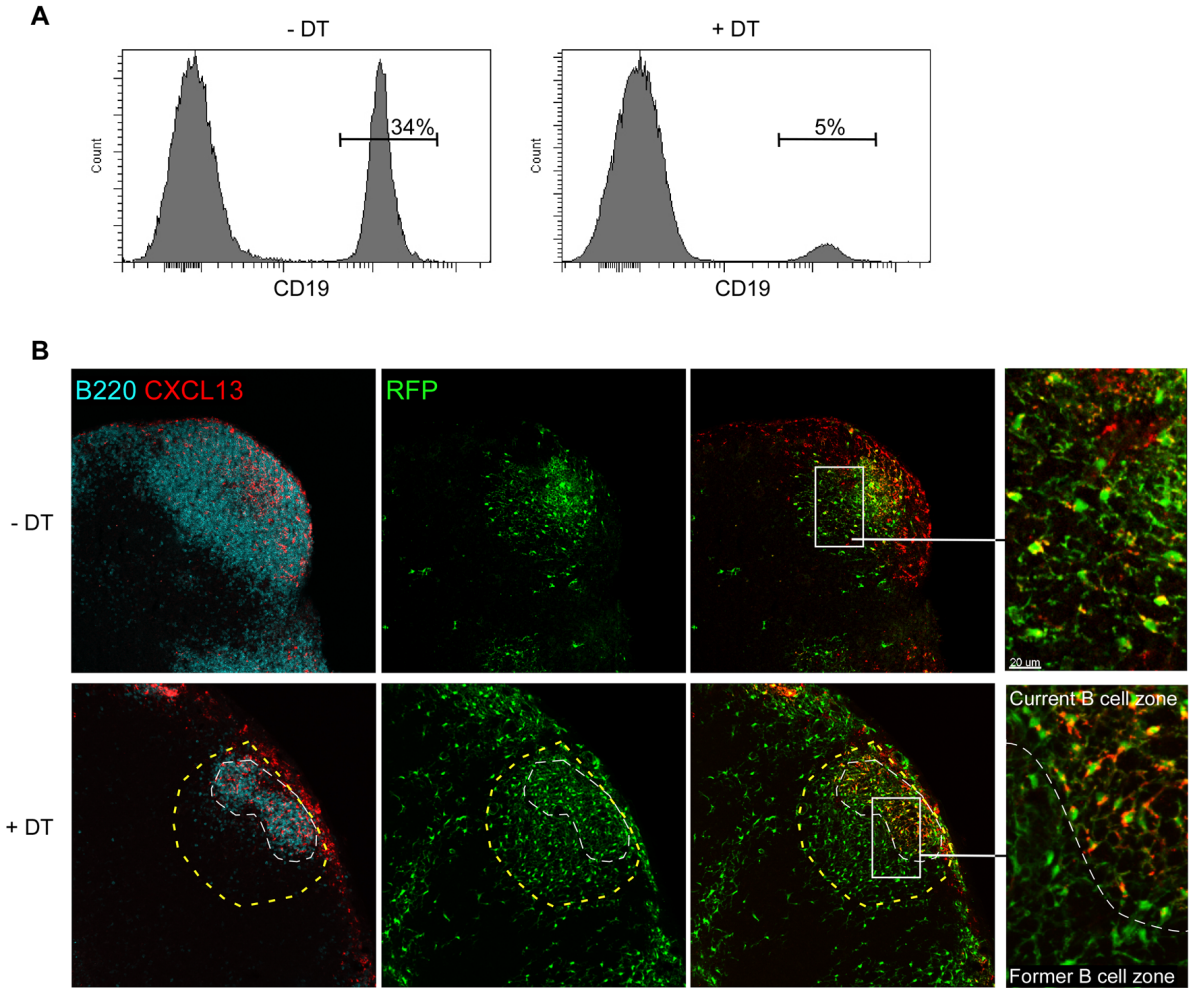
Acute B cell deletion abrogates CXCL13 staining in converted CD21^−^ RFP^+^ stromal cells. CD21cre-RFP mice were irradiated and reconstituted with bone marrow cells isolated from CD21cre-DTR mice. Reconstituted chimeras were injected with an emulsion of CFA/PBS in the ears. Three weeks later, mice were treated or not with DT for 3 consecutive days. The fourth day, ear draining LNs were harvested and either analyzed by flow cytometry (A) or sectioned and imaged by confocal microscopy (B). (A) Dot plots of ear draining LNs showing B cell deletion upon DT treatment. (B) Confocal images of LN tissue sections stained for B220 (blue) and CXCL13 expression (red). RFP^+^ cells appear in green. Inserts display high magnifications of the B cell areas. The yellow dashed line delineates the approximate size of the B cell follicle prior DT injection (see Materials and Methods), while the white dashed line delineates the boundary of the residual B cell follicle. Data are representative of three different experiments (two mice per experiment).

As B cells control the secretion of CXCL13 in FDCs, we sought to determine if the sudden accumulation of B cells around the CD21^−^ RFP^+^ stromal cells initially located in the T cell zone was responsible for their conversion. To this aim, CD21cre mice were crossed to the inducible Rosa-Diphteria Toxin Receptor (DTR) mice [29]. In these double Tg mice, all cells that express or have expressed CD21 display the DTR on their surface, rendering them susceptible to deletion following DT injection. CD21cre-RFP mice were irradiated and reconstituted with CD21cre-DTR bone marrow cells in order to obtain animals in which FDCs and the new subset of stromal cells would express RFP in absence of the DTR, while all mature colorless B cells would express the DTR. Such chimeric mice were injected with CFA in the ears, and 3 wk later, mice were treated or not with DT for 3 consecutive days. At that time, inflamed dLNs were harvested and either analyzed by flow cytometry or sectioned, stained for B220 and CXCL13, and analyzed by confocal microscopy. Flow cytometry analysis revealed that DT injection induced the ablation of ∼70%–90% of B cells in the LNs of treated mice (Figure 5A). In the LN sections of DT-treated chimeras, we determined that the remaining B cells were not evenly distributed in the areas formerly occupied by the enlarged B cell follicles but aggregated in small and compact B cell follicles centered around few CXCL13^+^ FDC-M2^+^ RFP^+^ cells (Figure 5B and unpublished data). This rapid withdrawal of the B cell follicles provided a unique opportunity to investigate if the sudden release from B cell control affected the capacity of converted CD21^−^ RFP^+^ stromal cells to secrete CXCL13. To this aim, we extrapolated the shapes that each inflamed B cell follicle approximately occupied before DT treatment on LN sections and counted the number of FDC-M2^−^ CXCL13^+^ and CXCL13^−^ RFP^+^ stromal cells present in these regions (see Materials and Methods, Figure 5, and Figure S6). Using this approach, we determined that only 12.9% (70 out of 543) of the RFP^+^ cells located in a region previously occupied by inflamed B cell follicles remained positive for CXCL13 expression (Figure 5B). These results demonstrated that B cells were responsible for the conversion and the maintenance of this new stromal cell type into CXCL13 secreting cells.

### LT-β Deficiency in B Cells Affects the Conversion of CD21^−^ RFP^+^ Stromal Cells

**Figure 6:**
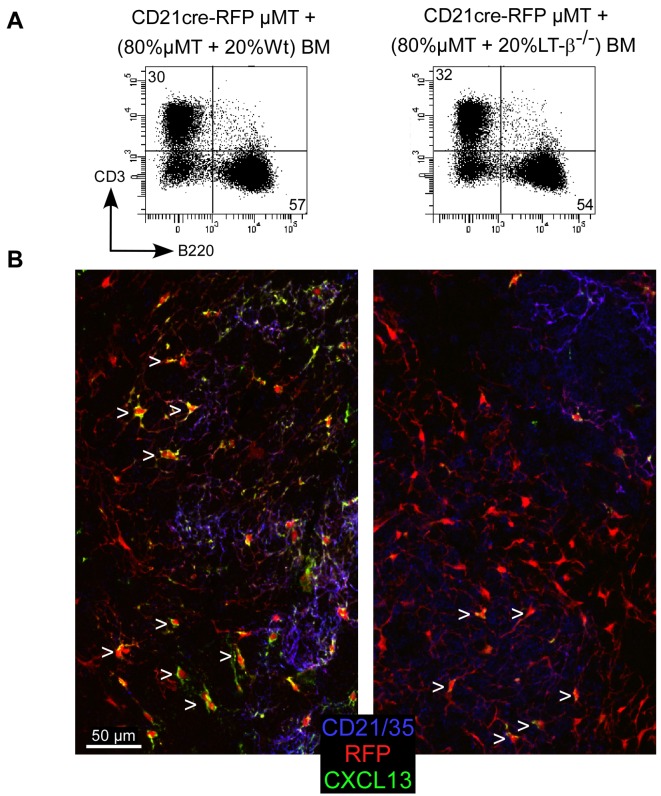
LT-β expression on B cells regulates CXCL13 expression in CD21^−^ RFP^+^ stromal cells. CD21cre-RFP μMt^−/−^ mice were irradiated and reconstituted with a mixture of μMt^−/−^ (80%) and Wt (20%) bone marrow cells or a mixture of μMt^−/−^ (80%) and LT-β–deficient bone marrow cells. Reconstituted chimeras were injected with an emulsion of CFA/PBS in the ears. Three weeks later, splenocytes were used to assess the percentage of CD3^+^ T cells and B220^+^ B cells in each group of mice by flow cytometry (A). Ear draining LNs were sectioned and stained for CD21/35 (blue), CXCL13 (green), and B220 expression (B). Confocal pictures were taken in B220^+^ B cell follicles. RFP^+^ cells appear in red. Arrowheads point to CXCL13^+^ CD21^−^ RFP^+^ stromal cells. Data are representative of two different experiments (three mice per experiment).

B lymphocytes are an essential source of membrane LT-β for establishing FDC networks and follicular organization via CXCL13 expression [20],[30]–[32]. As CXCL13 induces an up-regulation of LT-β in B cells, it creates a positive feedback loop that identifies LT-β expression on B cells as a key regulator of FDC development and CXCL13 secretion [8]. In order to assess if LT-β expression on B cells was responsible for the conversion of CD21^−^ RFP^+^ stromal cells in inflamed B cell follicles, we set up bone marrow chimeras in which B cells were deficient for LT-β expression. To this aim, irradiated CD21cre RFP μMT mice devoid of B cells were reconstituted with two different mixtures of bone marrow cells. Control mice received a mixture of μMT (80%) and Wt (20%) bone marrow cells. In these mice, all cells were LT-β sufficient. The other mice received a mixture of μMT (80%) and LT-β^−/−^ bone marrow cells, ensuring that all their B cells were deficient for LT-β expression. Flow cytometry analysis performed on the spleens of both groups of chimeras indicated a similar reconstitution of T and B cell compartments (Figure 6A). Chimeras were injected with CFA, and 3 wk later, their dLNs were sectioned; stained for B220, CD21, and CXCL13 expression; and analyzed by confocal microscopy. CD21 expression was used to differentiate CD21^+^ FDCs from CD21^−^ RFP^+^ stromal cells embedded in inflamed B cell follicles. Analysis of LN tissue sections revealed that 88% (264 cells out of 301) of CD21^−^ RFP^+^ cells displayed CXCL13 staining in chimeric mice reconstituted with LT-β–sufficient B cells. On the contrary, only 38% (195 out of 515) of CD21^−^ RFP^+^ cells were positive for CXCL13 staining in chimeric mice repopulated with LT-β–deficient B cells. In addition, the amounts of CXCL13 staining in such CD21^−^ RFP^+^ cells were decreased as compared to control animals (Figure 6B). These results suggested that the conversion of RFP^+^ stromal cells into CXCL13 secreting cells was modulated by B cells in a LT-β–dependant manner.

### Converted CD21^−^ RFP^+^ Stromal Cells Set Up the Boundaries of Inflamed B Cell Follicles

**Figure 7:**
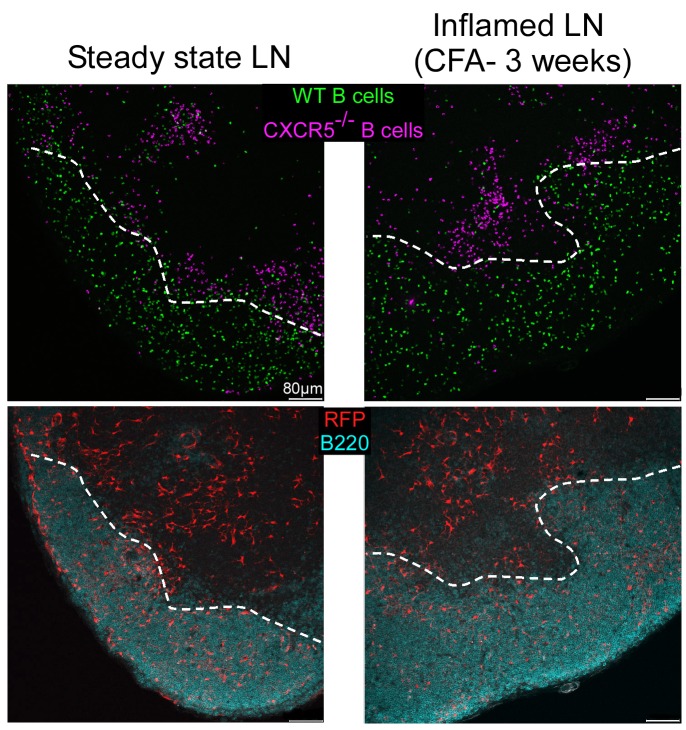
CXCR5-deficient B cells fail to access inflamed B cell follicles. CD21cre-RFP chimeras were untreated (left panel) or injected (right panel) with an emulsion of CFA/PBS in the ears. Three weeks later, recipients were injected with a cohort of CMFDA-labeled Wt-deficient B cells (green) and Celltrace violet–labeled CXCR5-deficient B cells (violet). One day later, ear draining LNs were sectioned, stained for B220 (blue) expression, and imaged by confocal microscopy. RFP^+^ cells appear in red. Data are representative of two different experiments (three mice per experiment).

GCs develop within the center of B cell follicles and are the location of an intense B cell proliferation, differentiation, and selection [25],[26]. During their expansion, GCs repel the naive B cells to their periphery, creating a characteristic follicular mantle continuously fueled by the recruitment of blood circulating naive B cells. Such massive immigration of naive B cells likely creates a need for B cell territorial expansion. As CXCL13 delineates the boundaries of the B cell follicles in noninflamed SLOs, we reasoned that, upon inflammation, the B cell–dependant induction of CXCL13 in the CD21^−^ RFP^+^ stromal cells located at the external border of the follicular mantle may create the additional B cell landmarks necessary for the expansion of B cell follicles. In order to test this hypothesis, we took advantage of mice deficient for CXCR5, the receptor of CXCL13 [8]. Naive B cells were purified from Wt and CXCR5-deficient mice, colored with different dyes, and co-injected in CD21cre-RFP chimeras injected 3 wk before with CFA in their ears. Ear dLNS and non-dLNs were harvested 1 d later, sectioned, and stained for B220 expression, and the capacity of Wt and CXCR5-deficient B cells to enter B cell follicles was assessed by confocal microscopy. Wt B cells were equally able to enter the B cell follicles of steady state and inflamed LNs ([8] and Figure 7). On the contrary, CXCR5-deficient B cells were unable to enter the B cell follicles of steady state and inflamed LNs and remained located in the T cell zone ([8] and Figure 7). These observations identify CXCL13 as the master regulator of naive B cell access to inflamed B cell follicles. As CXCR5-deficient B cells did not have access to CD21^−^ RFP^+^ cells that form large CXCL13 positive belts around more central FDCs (Figure 4), our results suggested that these CD21^−^ RFP^+^ cells “embedded” in B cell follicle borders delineated the boundaries of expanding inflamed B cell follicles. Further experiments will, however, be required to test the relative contribution of FDC- and CD21^−^ RFP^+^–derived CXCL13 in this process.

## Discussion

Using a reporter mouse that enables the fate mapping of CD21 expressing stromal cells, we discovered a stromal cell type that resides in the T cell zones of resting LNs and lacks the classical MRC, FDC, and preFDC hallmarks, including the secretion of CXCL13. In inflamed LNs, we demonstrated that activated B cell follicles progressively trespassed in the adjacent T cell zone, surrounded these stromal cells, and converted them via a LT-β pathway into CXCL13 secreting cells. Because of their unique plasticity, we have named these new stromal cells “VSCs” (versatile stromal cells).

To our knowledge, VSCs constitute the first example of plastic stromal cells able to adapt their secretion of chemokines according to their cellular environment. Importantly, the secretion of CXCL13 in converted VSCs is transient as demonstrated by the fact that VSCs initially located in the enlarged B cell follicles rapidly down-regulated their secretion of CXCL13 upon B cell follicle withdrawal. One could wonder why such B cell–dependent conversion of VSCs is not definitive once acquired. Upon the completion of an immune response, inflamed LNs return to homeostasis. During the cellular contraction that accompanies this event, T and B cell areas progressively return within their initial limits. As these limits are controlled by the secretion of CXCL13 and CCL21, maintaining a source of CXCL13 in the T cell zone of steady-state LNs may misguide incoming B cells [8],[9]. Blood circulating B cells enter the LNs via high endothelial venules (HEVs) located in the T cell zone [33]. Therefore, any CXCL13 secreting VSC of the T cell zone would be ideally located to retain B cells on their way to B cell follicles. Such misguidance may decrease the numbers of B cells in the follicles and hence impact the survival and sizes of FDC networks that in return may be impaired in their capacity to promote the survival, activation, and proliferation of B cells. By releasing their control on VSCs upon B cell follicle withdrawal, we believe that B cells ensure that VSCs no longer attract and retain B cells in the T cell zone of LNs that have returned to steady state.

B cells enter the LNs via HEVs located in the T cell zone and probably access the B cell follicles via its base/borders, precisely where CXCL13-secreting VSCs are located [34]. Our observation that adoptively transferred CXCR5-deficient B cells failed to enter inflamed B cell follicles suggests that converted VSCs create a belt of CXCL13 in charge of recruiting/retaining naive B cells into the follicular mantle. In this scenario, each new incoming B cell would enlarge the size of the follicular mantle and hence participate to the conversion of new “sleeper” VSCs into CXCL13^+^ B cell landmarks. Importantly, experimental data support the existence of a positive feedback loop between CXCL13 and LT-β [8]. In other words, B cells increase their expression of surface-bound LT-β when they encounter CXCL13. As HEVs continuously recruit massive numbers of blood-borne B cells in inflamed LNs, they would continuously fuel B cell follicles with naive B cells that will increase their level of LT-β expression upon their arrival in the follicle, allowing the induction of CXCL13 in recently surrounded VSCs.

One could wonder if the converted VSCs located at the border of the enlarged B cell follicles are the ones that were initially located in the T cell zone or if they derived from the FDCs located in the center of primary B cell follicles. Two lines of evidence favor the first assumption. First, unlike FDCs that require B cells to develop and survive, VSCs are present in the LNs of B cell–deficient mice, demonstrating that the two stromal cell types are different and that VSCs can develop in absence of FDCs [19],[20]. Second, regardless of their location and the nature of their surrounding lymphocytes (i.e., T or B cells), (i) the phenotype of resting and converted VSCs remains constant and different from the phenotype of MRCs, FDCs, and preFDCs [23] and (ii) VSCs—but not FDCs—remain attached to the conduit system normally restricted to the T cell zone. Altogether, these results suggest that converted VSCs in enlarged B cell follicles are not derived from FDCs but rather represent the population of VSCs initially located in the T cell zone of resting LNs.

In a recent article, Wang et al. used a CD21cre-Rosa DTR mouse to demonstrate the role of FDCs in establishing B cell follicle identity and promoting B cell retention in germinal centers [10]. As VSCs express RFP in CD21cre-Rosa-TdRFP reporter mice, there is little doubt that both FDCs and VSCs were depleted upon DT injection in CD21cre-Rosa DTR mice. Therefore, the relative contribution of FDCs and VSCs in the regulation of inflamed B cell follicles will require further experiments and the generation of new animal models.

Altogether, our results present VSCs as new functional members of the LN stromal cell family displaying unique properties. While the whole variety of their functions remains to be investigated, we propose that VSCs behave as transient surrogate FDCs in charge of accommodating the massive influx of naive B cells within inflamed B cell follicles.

## Materials and Methods

### Ethics Statement

All procedures performed on animals in this study have been approved by the ethical committee of the Université de la Méditerranée (France).

### Mice

C57BL/6J, B6.Cg-Tg(Cr2-cre)3Cgn/J (CD21/CR2-cre), B6.129S2(Cg)-*Cxcr5*tm1Lipp/J(CXCR5-deficient), B6-Gt(ROSA)26Sortm1(HBEGF)Awai (Rosa-DTR), and B6.129X1-*Gt(ROSA)*26Sortm1(EYFP)Cos/J (rosa YFP) mice were purchased from the Jackson Laboratory (Bar Harbor, ME); H. Luche and H. J. Fehling provided the rosa-RFP mouse [15]; and μMT mice were purchased from Cryopréservation Distribution Typage et Archivage animal (Orléans, France) and maintained in the CIML animal facilities. LT-β^−/−^ mice were originally produced at Yale University School of Medicine and have been maintained there since that time [35]. For the generation of chimeras, CD21cre-RFP mice were γ-irradiated (twice with 500 rads) from a cesium source and were reconstituted with various mixtures of bone marrow cells indicated in the main text (minimum of 2×10^6^ bone marrow cells per mouse). Chimeras were used at 8 wk postreconstitution after being tested for complete B cell chimerism.

### Antibodies

RA3-6B2 (anti-B220), 17A2 (anti-CD3 complex), 11-26c2a (anti-IgD), BP3 (anti CD157), and 2-4G2 (anti-CD16/32) antibodies were purchased from BD Biosciences Pharmingen (San Diego, California). APA5 (anti-CD140a/PDGFRα), 390 (anti-CD31/PECAM), 4E3 (anti-CD21/35), ALY7 (anti-Lyve-1), MDJ7/18 (anti-CD105), and IK22/5 (anti CD254/RANK-L) were purchased from e-bioscience (San Diego, California). BM-4018 (anti-ERTR-7) and polyclonal rabbit anti-Desmin antibodies were purchased from Acris Antibodies (Herford, Germany). AB19808 (anti-collagen IV) was purchased from abcam. Sc19982 (anti-VCAM-1) was purchased from Santa Cruz Inc (Santa Cruz, California). AF470 (anti-CXCL13) was purchased from R&D System (Minneapolis, Minnesota). 5H12 (anti-AIRE) and APB5 (anti-CD140b/PDGFR-β) were purchased from Biolegend (San Diego, California). FDC-M2 (anti-complement C4) was purchased from Immunokontact (Abingdon, UK). 18A2-G10 (anti-Mfge8) was purchased from MBL international (Woburn, MA). Anti-NG2 was purchased from Millipore (Billerica, MA), and RTD4E10 (anti-GP38/Podoplanin) was purchased from ReliaTech (Wolfenbüttel, Germany). LS17-9 (anti-thrombomodulin) was a gift from M. Aurrand-Lions. These antibodies were visualized by direct coupling to Pacific blue, allophycocyanin, Alexa fluor-488, -568, and -647, or through the use of alexa-fluor TSA kits or Alexa fluor -488, -568, -647, or -biotin coupled secondary antibodies (Life Technologies, Villebon sur Yvette, France).

### DT Treatment

Diphteria toxin was purchased from Calbiochem (Merck KGaA, Darmstadt, Germany). Mice were treated during 3 consecutive days (2 i.p. injections, 4 ng/g body weight) in order to efficiently delete B cells [29].

### Quantification of B Cell Follicle Regression Upon DT Treatment

LN immunofluorescence images were segmented into B220^+^ B cell areas in control and DT-treated chimeras. Such B cell areas were measured using Image J software (National Institutes of Health). The percentage of B cell follicle regression in DT-treated chimeras (as opposed to control mice) was calculated by dividing the total B cell area in control mice by the total B cell area in DT-treated mice. Of note, we observed a strict correlation between such ratio and the ratio of B cell percentages recorded by flow cytometry in the LNs of control and DT-treated mice. These ratios were then used to extrapolate the size that each B cell follicle occupied before DT treatment. As an example, if DT treatment induced a 70% reduction in the size of B cell follicles, we extrapolated that B cell follicles in DT-treated mice were 70% bigger before the treatment. We then drew a unique boundary on each B cell follicle of DT-treated mice (Figure 7).

### Immunostaining

LNs were harvested and fixed in a 0.05 M phosphate buffer containing 0.1 M L-lysine (pH 7.4), 2 mg/ml NaIO_4_, and 10 mg/ml paraformaldehyde (PLP) for 12 h, then washed in phosphate buffer and dehydrated in 30% sucrose in phosphate buffer. The 20 µm frozen sections were cut and then stained with the indicated antibodies as previously described [36]. Immunofluorescence confocal microscopy was performed with a Leica SP5 confocal microscope. Separate images were collected for each fluorochrome and overlaid to obtain a multicolor image. Final image processing was performed with Imaris software (Bitplane) and Adobe Photoshop.

### Flow Cytometry

LN cells were digested with collagenase I (Life Technologies, Villebon sur Yvette, France) for 30 min at 37°C and stained in 96-well V-bottomed plates. Cells were blocked with 2-4G2 for 15 min at 4°C and stained with antibodies in PBS 1% FCS 2 mM EDTA for 45 min at 4°C. Data were acquired on FACS-LSRII UV (Becton Dickinson) and were analyzed on DIVA software (Becton Dickinson).

### Cell Isolation/Sorting

LN stromal cells were isolated as previously described [23]. Pooled LNs from five mice were digested at 37°C in a digestion buffer (DMEM, 10 mM HEPES, penicillin/streptomycin, 1.2 mM CaCl2, 2% FCS) containing 1 mg/ml collagenase IV (Roche, Meylan, France) and 40 µg/ml DNase I (Roche) for 30 min. Aggregates were further digested (15 min) in a digestion buffer containing 3 mg/ml collagenase D (Roche) and 40 µg/ml DNase I. EDTA was added to a final concentration of 5 mM (pH 7.2) and cells were filtered through a 70 µm cell strainer. Cells were blocked in PBS containing 2% FCS, 2 mM EDTA, 2% mouse serum, and anti-CD16/32 antibody (2.4G2) stained and sorted (single round of sorting) with a FACS Aria III (85 µm nozzle). VSCs were sorted as CD31^−^CD21^−^ GP38^+^RFP^+^ cells and FRCs as CD45^−^CD31^−^CD21^−^ GP38^+^ RFP^−^ cells. The 5×10^4^ cells of each group were sorted directly in RLT buffer (QIAGEN, Courtaboeuf, France).

### Quantitative RT-PCR

Total RNA was extracted from sorted stromal cells using column-based system RNAeasy plus micro kit (QIAGEN) according to the manufacturer's instructions. Reverse-transcription was performed with quantitect Reverse Transcription kit (QIAGEN) and cDNAs analysed for CXCL13, CCL19, CCL21, and TGF-β1 transcripts by quantitative Real-Time PCR performed using SYBR Green PCRMaster Mix (QIAGEN) on an ABI-PRISM 7700 (Applied Biosystems) using default cycling conditions. Relative levels of target mRNA were compared with HPRT using the 2^−ΔΔCt^ method. The following primers were used: for CXCL13 sense, tggccagctgcctctctc; anti-sens, ttgaaatcactccagaacacctaca; for CCL19-atg sense, ctgcctcagattatctgccat; antisense, aggtagcggaaggctttcac; CCL21-ser sense, atcccggcaatcctgttctc; antisense, ggttctgcacccagccttc [7]; TGF-b1 sense, cctgagtggctgtcttttga; antisense, gtggagtacattatctttgctg; Adrb2 sense, gggaacgacagcgacttctt; and Adrb2 antisense, gccaggacgataaccgacat.

### Micro-Array Hybridization Data Normalization and Analysis

Biotinylated double-strand cDNA targets were prepared, starting from 0.3 to 3 ng of total RNA using the NuGEN Ovation Pico WTA System V2 Kit (Cat. No. 3302) and the NuGEN Encore Biotin Module Kit (Cat. No. 4200) according to NuGEN recommendations. Following fragmentation and endlabeling, 2 µg of cDNAs were hybridized for 16 h at 45oC on GeneChip Mouse Gene 1.0 ST arrays (Affymetrix) interrogating 28.853 genes represented by approximately 27 probes spread across the full length of the gene. The chips were washed and stained in the GeneChip Fluidicsstation 450 (Affymetrix) and scanned with the GeneChip Scanner 3000 7G (Affymetrix) at a resolution of 0.7 µm. Raw data (.CEL Intensity files) were extracted from the scanned images using the Affymetrix GeneChip Command Console (AGCC) version 3.2. Normalization of the raw Affymetrix expression data was performed by Robust Multi-chip Analysis (RMA) through Bioconductor (release 2.9) in the R statistical environment (version 2.14) via the Affy package. Probesets over a normalized expression value of 4 (log_2_ scale) across all arrays were considered as expressed and were included in the analysis. ProbeSets with a standard deviation >1 for each condition and that were not associated with a gene symbol were filtered out. At the end, 14,430 probes were kept for the subsequent analyses. In order to select the regulated probes between conditions, we used limma package (Linear Model for Microarray Data) using an empirical Bayes method. Genes having a log_2_ ratio superior to 1.5 and a *p* value inferior to 0.05 was considered as up-regulated in CD21^−^ RFP^+^ cells. The data discussed in this publication have been deposited in NCBI's Gene Expression Omnibus and are accessible through GEO Series Accession Number GSE47906 (http://www.ncbi.nlm.nih.gov/geo/query/acc.cgi?acc=GSE47906).

## Supporting Information

Figure S1 **CD21^−^ RFP^+^ stromal cells develop in absence of B and T cells.** (A) Comparison of LN sections from a CD21cre-RFP chimera and a CD21cre-RFP μMT mouse. LNs were stained for Collagen IV (white), CD3 (dark blue), and B220 (light blue) expression and analyzed by confocal microscopy. RFP^+^ cells appear in red. Inserts display high magnifications of the T cell area. (B) Confocal image of a LN section from a CD21cre-RFP RAG2^−/−^ mouse stained for Collagen IV expression (white). (C) CMFDA-labeled WT polyclonal B cells were injected in a CD21cre-RFP μMT mouse. The LNs of the recipient mouse were harvested and imaged 1 d later by confocal microscopy. Data are representative of two different experiments (two mice per experiment).
(TIF)

Figure S2 **CD21^−^ RFP^+^ stromal cells are embedded in the FRC network.** CD21cre-RFP Ubiquitin-GFP RAG2^−/−^ mice were irradiated and reconstituted with Wt bone marrow cells. In these chimeras, all LN stromal cells expressed GFP and hence appeared green, while CD21^+^ RFP^+^ FDCs and CD21^−^ RFP^+^ cells also co-expressed RFP and thus appeared orange [4]. LN sections isolated from such chimeric mice were stained for Collagen IV (white) and CD21 (blue) expression. GFP^+^ RFP^−^ expressing cells appear green, while GFP^+^ RFP^+^ expressing cells appear orange. Note how CD21^−^ GFP^+^ RFP^+^ cells are “embedded” in the CD21^−^ GFP^+^ RFP^−^ FRC network of the T cell zone. Data are representative of two different experiments (two mice per experiment).
(TIF)

Figure S3 **Kinetics of LN B cell recruitment following CFA/PBS injection.** Wt mice were injected with an emulsion of CFA/PBS in the ears. Ear draining LNs were harvested at the indicated times and analyzed by flow cytometry in order to determine the absolute numbers of B cells, CD8^+^ T cells, and CD4^+^ T cells present in the ear draining LNs of the mice. Data are representative of two different experiments (three mice per time point).
(TIF)

Figure S4 **Inflamed B cell follicles trespass in the adjacent T cell zone.** Mice were injected or not with an emulsion of CFA/PBS in the ears. Three weeks later, ear draining LNs were sectioned; stained for CD3 (blue), B220 (red), and collagen-IV expression (white); and imaged by confocal microscopy. The dashed lines delineate T/B boundaries areas, while the arrow indicates the collagen-enriched area of the inflamed B cell follicle. IR, Interfollicular Region. Data are representative of three different experiments (two mice per experiment).
(TIF)

Figure S5 **CD21^−^ RFP^+^ stromal cells are “annexed” by Inflamed B cell follicles.** CD21cre-RFP chimeras were injected with an emulsion of CFA/PBS in the ears. Three weeks later, ear draining LNs were sectioned; stained for PDGFRβ (green), B220 (blue), and collagen-IV expression (white); and imaged by confocal microscopy. RFP^+^ cells appear in red. Note how the central part of the follicle (*) that contains sparse conduits is populated by PDGFRβ^lo^ FDCs, while the inner border of the follicles enriched in conduits (arrows) contains numerous PDGFRβ^hi^ RFP^+^ cells. The dashed line represents the delineation of the B220 staining. Data are representative of two different experiments (two mice per experiment).
(TIF)

Figure S6 **Quantification of B cell follicle regression upon DT treatment.** LN immunofluorescence images were segmented into B220^+^ B cell areas in control and DT-treated chimeras. The percentage of B cell follicle regression in DT-treated chimeras (as opposed to control mice) was calculated by dividing the total B cell area in control mice by the total B cell area in DT-treated mice. These ratios were then used to extrapolate the size that each B cell follicle occupied before DT treatment. As an example, if DT treatment induced a X% reduction in the size of B cell follicles, we extrapolated that B cell follicles in DT-treated mice were X% bigger before the treatment and drew a corresponding boundary.
(TIF)

## Abbreviations

CFA: Complete Freund Adjuvant
dLN: draining Lymph Node
DT: diphteria toxin
DTR: diphteria toxin receptor
FDC: follicular dendritic cell
FRC: fibroblastic reticular cell
GC: germinal center
HEV: high endothelial venule
LN: lymph node
LTβ: lymphotoxin β
Mfge8: milk-fat globule epidermal growth factor 8
MRC: marginal reticular Cell
PDGF-Rα or β: platelet derived growth factor receptor
RANK-L: receptor activator of nuclear factor kappa-β ligand
RT-PCR: real-time polymerase chain reaction
SLO: secondary lymphoid organ
tdRFP: tandem dimer Red Fluorescent Protein
VSC: versatile stromal cell
Wt: wild type
YFP: yellow fluorescent protein
