# Supplementary figures and images for: Identification of a New Stromal Cell Type Involved in the Regulation of Inflamed B Cell Follicles
Source: PLoS Biol. 2013 Oct 1;11(10):e1001672. doi: 10.1371/journal.pbio.1001672 (PMC3794863; doi:10.1371/journal.pbio.1001672)

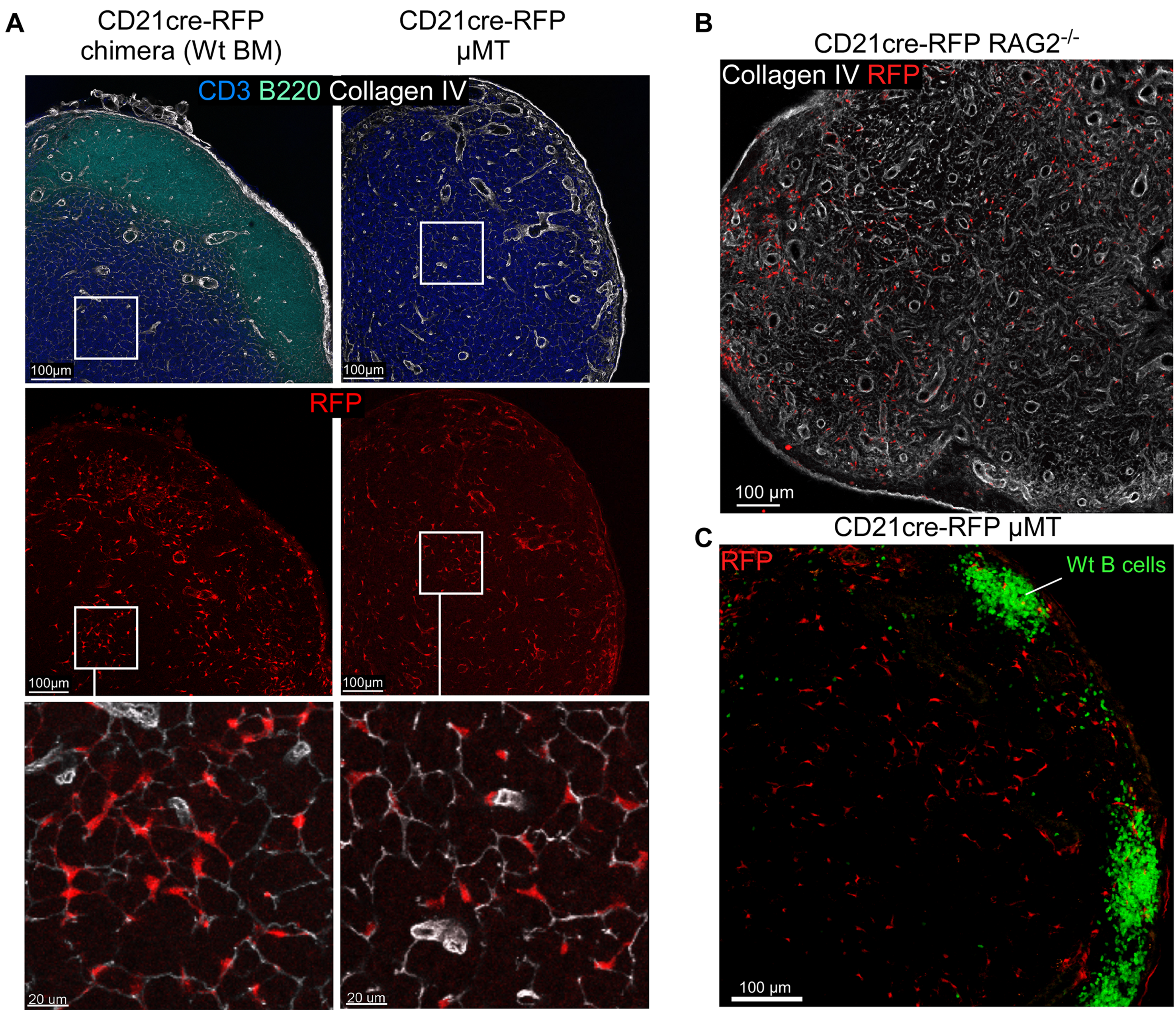

Supplement: Figure S1 — CD21− RFP+ stromal cells develop in absence of B and T cells. (A) Comparison of LN sections from a CD21cre-RFP chimera and a CD21cre-RFP μMT mouse. LNs were stained for Collagen IV (white), CD3 (dark blue), and B220 (light blue) expression and analyzed by confocal microscopy. RFP+ cells appear in red. Inserts display high magnifications of the T cell area. (B) Confocal image of a LN section from a CD21cre-RFP RAG2−/− mouse stained for Collagen IV expression (white). (C) CMFDA-labeled WT polyclonal B cells were injected in a CD21cre-RFP μMT mouse. The LNs of the recipient mouse were harvested and imaged 1 d later by confocal microscopy. Data are representative of two different experiments (two mice per experiment). (TIF) [file pbio.1001672.s001.tif]

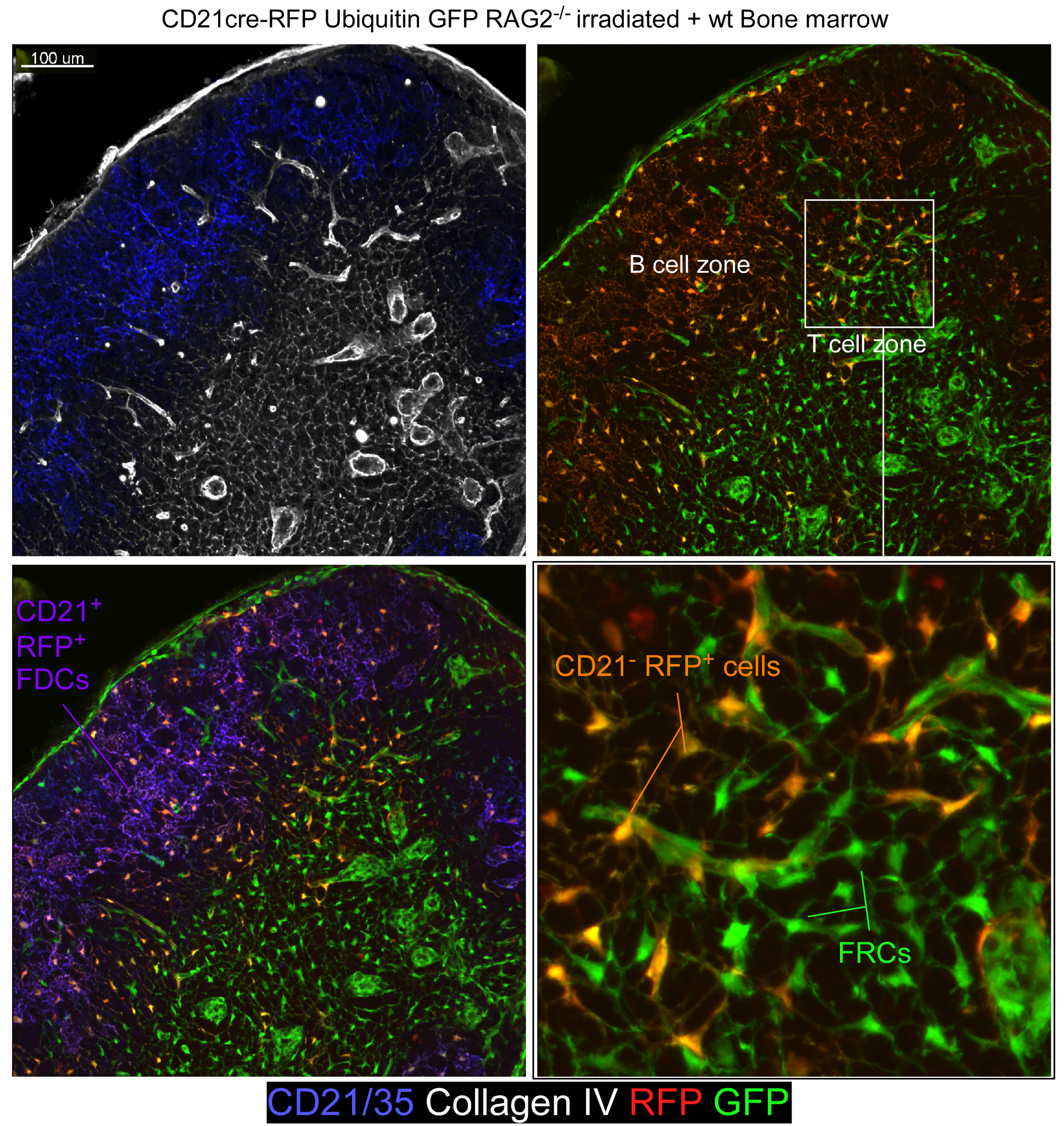

Supplement: Figure S2 — CD21− RFP+ stromal cells are embedded in the FRC network. CD21cre-RFP Ubiquitin-GFP RAG2−/− mice were irradiated and reconstituted with Wt bone marrow cells. In these chimeras, all LN stromal cells expressed GFP and hence appeared green, while CD21+ RFP+ FDCs and CD21− RFP+ cells also co-expressed RFP and thus appeared orange [4]. LN sections isolated from such chimeric mice were stained for Collagen IV (white) and CD21 (blue) expression. GFP+ RFP− expressing cells appear green, while GFP+ RFP+ expressing cells appear orange. Note how CD21− GFP+ RFP+ cells are “embedded” in the CD21− GFP+ RFP− FRC network of the T cell zone. Data are representative of two different experiments (two mice per experiment). (TIF) [file pbio.1001672.s002.tif]

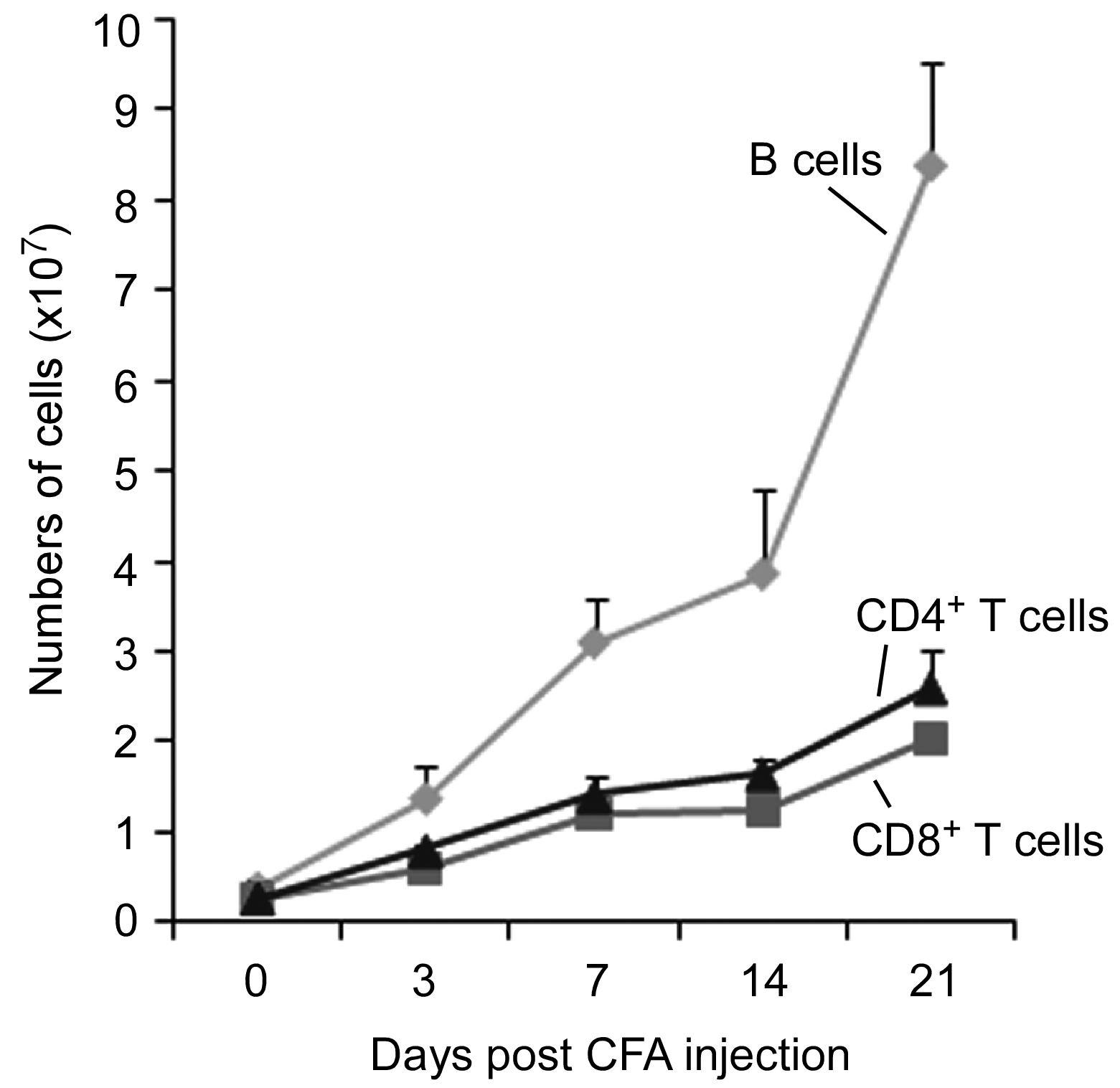

Supplement: Figure S3 — Kinetics of LN B cell recruitment following CFA/PBS injection. Wt mice were injected with an emulsion of CFA/PBS in the ears. Ear draining LNs were harvested at the indicated times and analyzed by flow cytometry in order to determine the absolute numbers of B cells, CD8+ T cells, and CD4+ T cells present in the ear draining LNs of the mice. Data are representative of two different experiments (three mice per time point). (TIF) [file pbio.1001672.s003.tif]

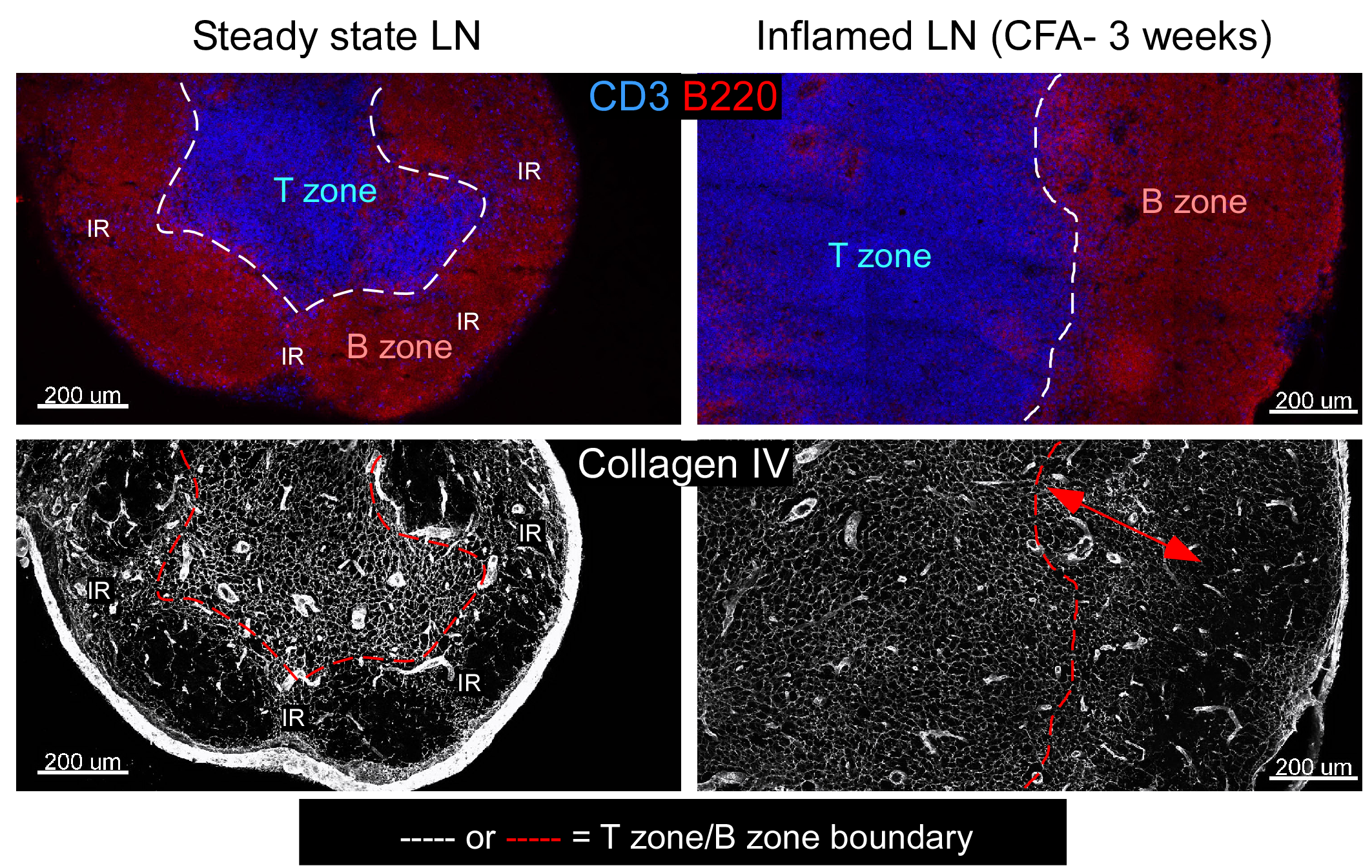

Supplement: Figure S4 — Inflamed B cell follicles trespass in the adjacent T cell zone. Mice were injected or not with an emulsion of CFA/PBS in the ears. Three weeks later, ear draining LNs were sectioned; stained for CD3 (blue), B220 (red), and collagen-IV expression (white); and imaged by confocal microscopy. The dashed lines delineate T/B boundaries areas, while the arrow indicates the collagen-enriched area of the inflamed B cell follicle. IR, Interfollicular Region. Data are representative of three different experiments (two mice per experiment). (TIF) [file pbio.1001672.s004.tif]

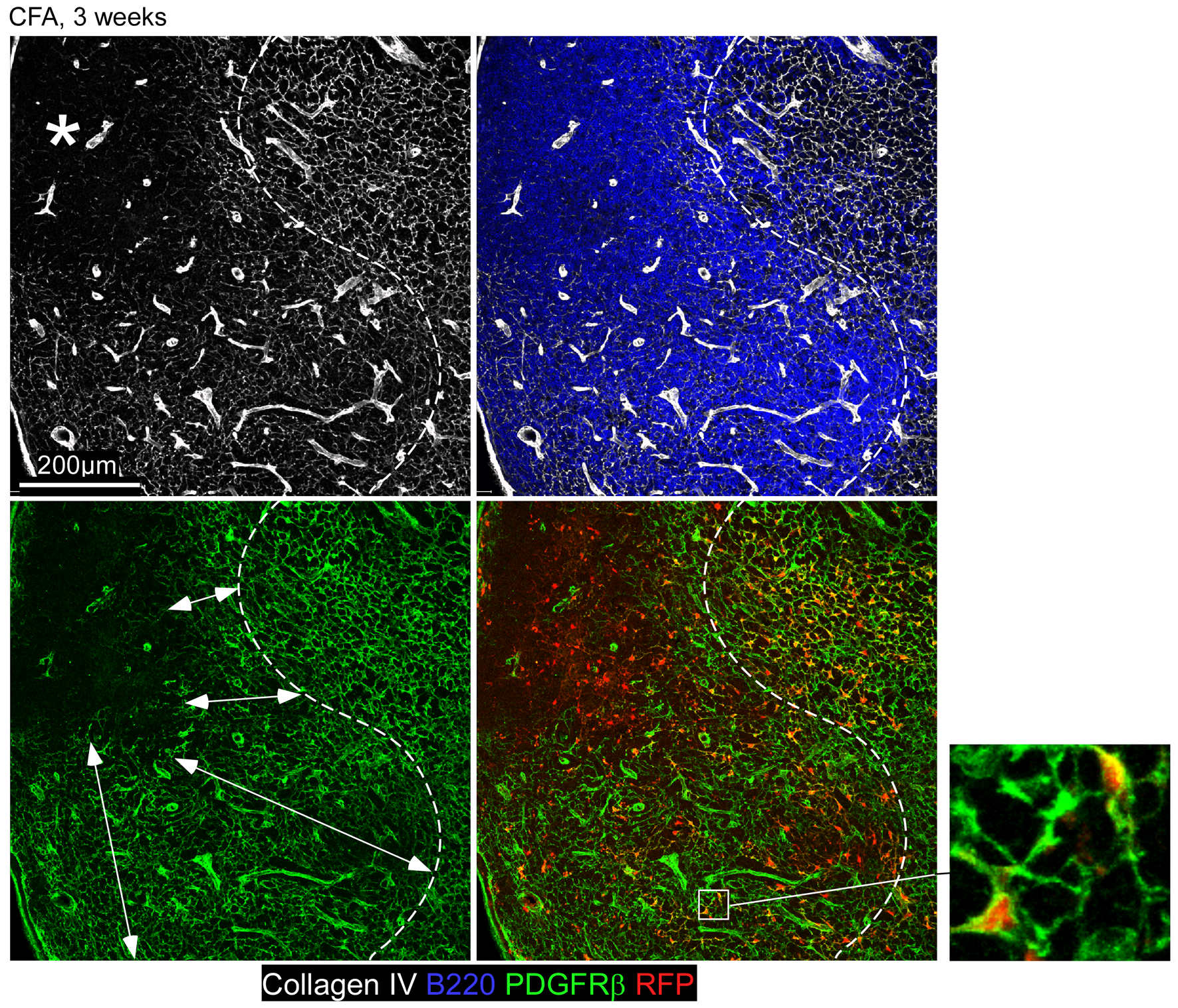

Supplement: Figure S5 — CD21− RFP+ stromal cells are “annexed” by Inflamed B cell follicles. CD21cre-RFP chimeras were injected with an emulsion of CFA/PBS in the ears. Three weeks later, ear draining LNs were sectioned; stained for PDGFRβ (green), B220 (blue), and collagen-IV expression (white); and imaged by confocal microscopy. RFP+ cells appear in red. Note how the central part of the follicle (*) that contains sparse conduits is populated by PDGFRβlo FDCs, while the inner border of the follicles enriched in conduits (arrows) contains numerous PDGFRβhi RFP+ cells. The dashed line represents the delineation of the B220 staining. Data are representative of two different experiments (two mice per experiment). (TIF) [file pbio.1001672.s005.tif]

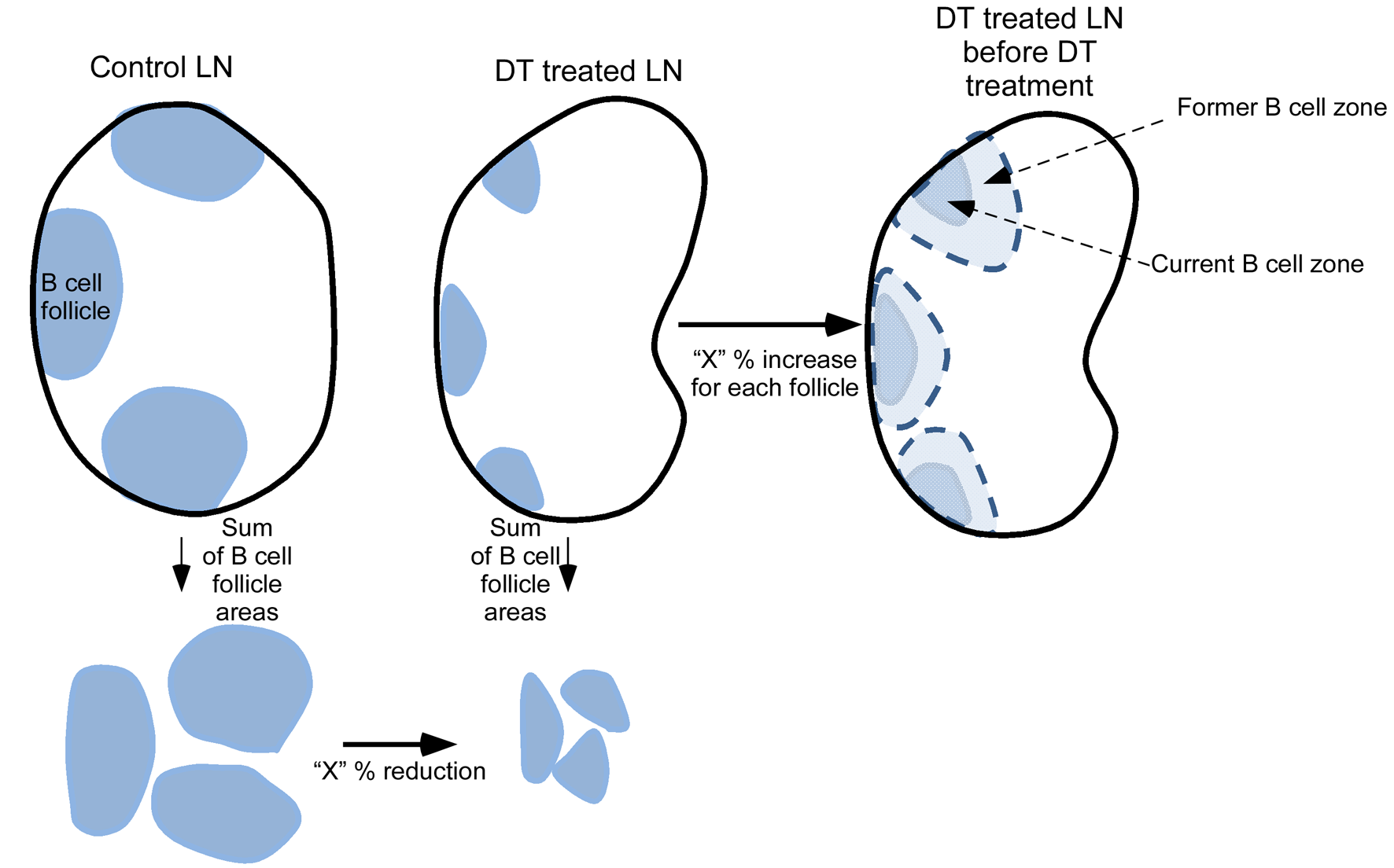

Supplement: Figure S6 — Quantification of B cell follicle regression upon DT treatment. LN immunofluorescence images were segmented into B220+ B cell areas in control and DT-treated chimeras. The percentage of B cell follicle regression in DT-treated chimeras (as opposed to control mice) was calculated by dividing the total B cell area in control mice by the total B cell area in DT-treated mice. These ratios were then used to extrapolate the size that each B cell follicle occupied before DT treatment. As an example, if DT treatment induced a X% reduction in the size of B cell follicles, we extrapolated that B cell follicles in DT-treated mice were X% bigger before the treatment and drew a corresponding boundary. (TIF) [file pbio.1001672.s006.tif]
